# Use of the Slow-Delivery Platform, VacSIM, Shapes the Host Immune Response to Increase Protection Against Influenza Infection

**DOI:** 10.3390/v17091190

**Published:** 2025-08-30

**Authors:** Anna L. McCormick, Ted M. Ross, Donald A. Harn, Jarrod J. Mousa

**Affiliations:** 1 Center for Vaccines and Immunology, College of Veterinary Medicine, University of Georgia, Athens, GA 30602, USA; anna.mccormick@med.fsu.edu (A.L.M.); rosst7@ccf.org (T.M.R.); 2 Department of Infectious Diseases, College of Veterinary Medicine, University of Georgia, Athens, GA 30602, USA; dharn@uga.edu; 3 Department of Biomedical Sciences, College of Medicine, Florida State University, Tallahassee, FL 32304, USA; 4 Florida Research and Innovation Center, Cleveland Clinic Florida, 9801 SW Discovery Way, Port Saint Lucie, FL 34986, USA; 5 Department of Infection Biology, Lerner Research Institute, Cleveland Clinic, Cleveland, OH 44195, USA

**Keywords:** influenza, vaccine, COBRA, hemagglutinin, slow-delivery, antibody

## Abstract

Influenza virus is a leading cause of global morbidity and mortality due to acute lower respiratory infection, even with the widespread use of multiple licensed influenza vaccines. However, antigenic drift during influenza replication can cause vaccine-induced antibodies to poorly neutralize influenza virus, thereby reducing vaccine effectiveness. To help overcome this problem, we leveraged a hydrogel platform with influenza hemagglutinin (HA) protein to induce prolonged antigen exposure. The hydrogel platform, Vaccine Self-Assembling Immune Matrix (VacSIM^®^), in combination with recombinant influenza H1 or H3 HA protein antigens, increased antigen-specific antibody titers in vaccinated mice, which led to decreased disease severity after H1N1 infection for H1 HA-vaccinated mice and decreased lung viral titers after H3N2 challenge for H3 HA-vaccinated mice. Sera collected from mice immunized with VacSIM and HA also showed broader HAI activity, increasing by 1–3 log against a panel of influenza viruses. These results were consistent with the use of cocktail immunization, containing both an H1 and H3 HA, where mice immunized with VacSIM had an increase in antigen-specific antibody titers and decreased disease severity and lung viral titers against H1N1 and H3N2 influenza challenges, respectively. Finally, it was determined that a single immunization with VacSIM and H1 HA could provide protection against lethal H1N1 challenge compared to a group without VacSIM. In summary, we demonstrate that use of the slow-release platform VacSIM can improve the host immune response to vaccination and increase protection against influenza infection.

## 1. Introduction

Influenza virus is one of the leading causes of global morbidity and mortality due to respiratory infection and poses a significant public health burden [1,2]. To lessen this disease burden, there are multiple licensed seasonal influenza vaccines [3]. However, current seasonal influenza vaccines often do not provide complete protection against circulating strains of influenza virus, as annual vaccination elicits variable levels of protection between peak influenza seasons and is short-lived [4,5]. Current seasonal influenza vaccines elicit antibodies targeting the surface proteins found on the virus, with the antibody response primarily targeting the hemagglutinin (HA) protein [4,6]. The HA protein is highly immunogenic and is the major target for protective antibodies in currently approved vaccines [7,8]. The HA protein is important in early stages of the viral life cycle as it binds to sialic acid found on host cells, allowing the virus to enter the cell via endocytosis to initiate infection [8,9]. HA is more abundant on the surface of the virus than other surface proteins, such as neuraminidase, and is immunodominant [4,10]; however, the highly mutable nature of hemagglutinin leads to varying levels of vaccine-elicited protection [5].

Vaccination against influenza virus is the most effective countermeasure against influenza disease. There are three classes of influenza vaccines that are offered each year, which include live-attenuated, split-inactivated, and recombinant HA vaccines [6,11]. All of these vaccines contain multiple influenza viruses or proteins, representing both influenza A (H1N1 and H3N2) as well as influenza B (Yamagata and/or Victoria lineages), creating a trivalent or quadrivalent vaccine [12,13]. However, due to antigenic drift, where the error-prone RNA-dependent RNA polymerase of influenza virus causes accumulation of mutations within the HA protein and other proteins, vaccine-elicited antibodies from previous years may be unable to recognize newly mutated HA proteins [5,14]. Additionally, antigenic shift, the genetic reassortment of two or more influenza viruses in a host leading to production of new viruses, can result in poor preexisting antibody recognition of influenza virus [15]. Antigenic shift is facilitated by the viral segmented genome, allowing for entire gene segments to be exchanged when two different viruses infect the same cell [15]. This process can create novel genotypes of influenza viruses with hemagglutinin (HA) and neuraminidase (NA) proteins that are unrecognizable to existing vaccine-induced anti-influenza immunity. Both antigenic drift and antigenic shift pose significant obstacles for current and future influenza vaccines, as long-lasting protection against an ever-changing influenza virus proves to be difficult. To overcome this problem, many novel influenza vaccines are in preclinical and clinical development, specifically to induce broadened immunity and longer-lasting protection. These next generation vaccines include both improved antigen formulation and new mechanisms of antigen delivery to the host. One of these vaccine design platforms, computationally optimized broadly reactive antigen (COBRA) [16], aligns several wildtype HA or NA sequences over a select time period to obtain a single consensus sequence that can provide broader vaccine-elicited antibody breadth against influenza virus [16,17]. Vaccination with COBRA HA and NA antigens has demonstrated increased breadth of vaccine-elicited antibodies as well as improved protection against subsequent influenza challenge [11,16,17,18].

Other researchers have studied usage of delivery platforms that can introduce the antigen to the host in alternative ways [19,20]. Emerging platforms for vaccine delivery include nanoparticle platforms, mRNA lipid nanoparticle vaccines, and viral-vectored vaccines [21]. Many of these novel delivery platforms focus on extended release or production of antigen within host cells [19,20,22]. Extended release of antigen increases the duration of the immune response, leading to higher antigen-specific antibody titers and increased breadth [22,23]. The exact mechanism as to how extended antigen release facilitates an improved immune response remains to be determined. In this study, we aimed to determine the effects of using a slow delivery platform called Vaccine Self-Assembling Immune Matrix, or VacSIM. VacSIM is a formulation of the (RADA)_4_ peptide initially described by Zhang et al. [24] where the peptide contains alternating hydrophobic and hydrophilic residues that allow it to assemble into higher-order 3D structures [25]. The (RADA)_4_ formulation is used as a commercially available hemostat, also known as Purastat^TM^, which is commonly used in gastrointestinal surgeries to clot bleeding and has been shown to be non-immunogenic in humans [26]. Previous work has demonstrated that the (RADA)_4_ peptide will self-assemble into a hydrogel with 50–200 nm pores under physiological conditions, resembling the structure of natural collagen [27]. VacSIM works as a “plug and play” matrix where vaccine components are mixed with the peptides and can be used immediately, making the use of VacSIM a versatile and promising slow-delivery platform. Preliminary studies using VacSIM as a delivery platform have demonstrated an increase in antigen-specific antibody titers for both influenza and hepatitis B vaccination [28,29].

In this study, we determined the effect of VacSIM on the vaccine-elicited antibody response to two COBRA antigens. We analyzed the titers and breadth of vaccine-elicited antibodies as well as protection observed after subsequent homologous influenza infection for both the H1 and H3 subtypes of HA with or without the use of the delivery platform VacSIM. We found that use of VacSIM, in addition to different COBRA HA proteins, increased antigen-specific antibody titers for both the H1 COBRA antigen, Y2, generated from H1 sequences from the years 2014–2016, and the H3 COBRA antigen, NG2, generated from H3 sequences from the years 2016–2018. Similarly, we observed that vaccination with VacSIM led to increased antibody breadth and functionality when combined with the COBRA antigens. Overall, these data suggest that use of the slow delivery platform VacSIM plays a significant role in increasing functional antibody titers as well as protection against subsequent influenza infection.

## 2. Materials and Methods

### 2.1. Ethics Statement

All procedures were reviewed and approved by the University of Georgia Institutional Animal Care and Use Committee (IACUC). Animals were rested for 3 days before experiments commenced and were fed ad libitum.

### 2.2. Protein Purification

Trimeric Y2 and NG2 COBRA HA proteins were expressed and purified in Expi293F cells and verified by Western blot as previously described [18]. Plasmids for both Y2 and NG2 COBRA HA proteins were transformed in DH5a *E. coli* cells, and the bacteria containing the Y2 or NG2 plasmids were maxiprepped (D6922-04, Omega Biotech, Norcross, GA, USA) following the manufacturer’s protocol. These plasmids were transfected into Expi293F cells and the collected supernatants containing either the Y2 or NG2 antigens were purified on a HisTrap Excel column (17371206, Cytiva, Marlborough, MA, USA), following the manufacturer’s protocol. Each elution containing purified HA protein was verified for antigen integrity by SDS-PAGE and Western blot by probing with anti-HIS tag (clone J099B12, BioLegend, San Diego, CA, USA) and anti-HA antibodies (clone 34C9; Immune Technology Corp, New York, NY, USA) as previously described [30]. Once purified, these proteins were stored in the −80 °C freezer until further use.

### 2.3. Virus Growth and Titering

The H1N1 influenza viruses used, A/California/07/2009, A/Brisbane/59/2007, A/Brisbane/02/2018, and A/Guangdong-Maonan/SWL1536/2019 were grown and passaged in Madin-Darby Canine Kidney (MDCK) London (FR-58, IRR, Manassas, VA, USA) cells for 48 hrs in virus growth media (VGM: DMEM with L-glutamine (10-013-CM, Corning, Corning, NY, USA), 7.5% Bovine Serum Albumin (BSA) (BP9700100, Thermofisher, Waltham, MA, USA), 1 µg /mL TPCK-Trypsin (20233, Thermofisher)). Prior to infection, MDCK London cells were passaged in MDCK London growth media (DMEM with L-glutamine (10-013-CM, Corning, Corning, NY, USA), 10% FBS (A5256701, Thermofisher, Waltham, MA, USA), 1% antibiotic-antimycotic (15240062, Thermofisher, Waltham, MA, USA)). The H3N2 viruses used, A/Brisbane/10/2007, A/Switzerland/9715293/2013, and A/Kansas/14/2017, were grown and passaged in MDCK SIAT-1 cells for 48 hrs in VGM. Prior to infection, MDCK SIAT-1 cells were passaged in MDCK SIAT growth media (DMEM with L-glutamine (10-013-CM, Corning, Corning, NY, USA), 10% FBS (A5256701, Thermofisher, Waltham, MA, USA), 2% geneticin (10-131-035, Thermofisher, Waltham, MA, USA)). All viruses were titered through hemagglutination assays (HA) as described below. Viruses were also titered through focal forming assays (FFA) [31]. For virus titration, both MDCK London (for H1N1 viruses) and MDCK SIAT-1 (for H3N2 viruses) cells were plated at a concentration of 4.8 × 10^6^ cells/mL (6 × 10^5^ cells per well) in 96-well plates and grown at 37 °C with 5% CO_2_ for 24 h, until confluent. The cells were washed twice with PBS. Following the wash, 50 µL of virus growth media (VGM: DMEM with L-glutamine (10-013-CM, Corning, Corning, NY, USA), 7.5% Bovine Serum Albumin (BSA) (BP9700100, Thermofisher, Waltham, MA, USA), 1 µg /mL TPCK-Trypsin (20233, Thermofisher, Waltham, MA, USA)) was added to each well, and the plate was incubated at 37 °C until ready for use. The virus stocks were diluted 10-fold in VGM across a 96-well plate. 50 µL of each dilution was added to the MDCK SIAT-1 cells and incubated at 37 °C for 2 h. After 2 h, an overlay was added to the cells (containing DMEM (MT90013PB, Corning, Corning, NY, USA), 0.1% BSA (BP9700100, Thermofisher, Waltham, MA, USA), 1.2% Avicel (11-101-5796, Thermofisher, Waltham, MA, USA), 1 µg/mL TPCK-Trypsin (20233, Thermofisher), 2.4% cellulose (AAA1773036, Thermofisher, Waltham, MA, USA)) and placed back in the 37 °C incubator overnight. The following day, the cells were washed twice with PBS and fixed for 30 min with 20% acetone/80% methanol at 4 °C, then subsequently washed. Following the wash, the plates were incubated with permeabilization buffer (glycine in 1X PBS (G46-500, Thermofisher, Waltham, MA, USA), 5% Triton X-100 (AAA16046AE, Thermofisher, Waltham, MA, USA)) for 30 min at room temperature and washed 2× with 0.05% PBS-Tween-20. A mouse polyclonal antibody (FR-1217, IRR, Manassas, VA, USA) targeting the nucleoprotein of the influenza virus was diluted 1:5000 in FRA ELISA buffer (10% heat-inactivated goat serum (16-210-072, Thermofisher, Waltham, MA, USA), 0.01M PBS, 0.1% Tween-20 (AAJ20605AP, Thermofisher, Waltham, MA, USA)) for 1 h at room temperature and then washed 2× with 0.05% PBS-Tween-20. Goat anti-mouse IgG Fc-HRP secondary antibody (1030-05, Southern Biotech, Birmingham, AL, USA), diluted 1:4000 in FRA ELISA buffer, was added and incubated for 1 hr at room temperature. The plates were washed 3× with 0.05% PBS-Tween-20. Afterwards, TrueBlue peroxidase (5510-0030, SeraCare^TM^, Milford, MA, USA) was added for 15 min in the dark at room temperature. The plates were washed with ddH_2_O, allowed to dry, and read on a CTL ImmunoSpot machine (Immunospot by C.T.L, Shaker Heights, OH, USA).

### 2.4. Hemagglutination Assays (HAs)

For the HAs, 50 µL of virus was diluted two-fold in PBS following a 1:2 dilution scheme for 11 dilutions (1:2 to 1:2048). Turkey whole blood in Alsevers’ solution (Lampire, Pipersville, PA, USA) (used for H1N1 viruses) or guinea pig whole blood in Alsevers’ solution (Lampire, Pipersville, PA, USA) (used for H3N2 viruses) was washed three times in PBS and diluted to a starting concentration of 0.8% in PBS. 50 µL of the 0.8% turkey or guinea pig red blood cells were added to the diluted virus in each well. Plates were incubated at room temperature for 30 min, and the well with the highest virus dilution that did not cause the agglutination of the red blood cells was the HA titer used for the HAI experiments.

### 2.5. Influenza Challenge Readouts

Prior to the start of the vaccination study, a lethal dose study was performed with five-to-six week old female DBA/2J mice (*n* = 5 mice per group) (Jackson Laboratory, Bar Harbor, MA, USA). Mice were infected with either 10^2^, 10^3^, or 10^4^ FFU of A/California/07/2009 H1N1 to determine the lethal dose that was used in future survival challenges. All doses caused 100% mortality in the mice, and 10^3^ FFU was chosen as the dose used for subsequent challenges to create a stringent infection model at a minimum 10× above the lethal dose of 10^2^. For each H1N1 influenza challenge, mice were administered 10^3^ FFU of A/California/07/2009 (12.5 µL of a 1 × 10^7^ FFU/mL stock in 5 mL PBS) through an intranasal route (40 µL total, 20 µL each nostril) 56 days post-prime and monitored over the course of 14 days post-infection. The mice were monitored for the presence of labored breathing and lethargy as well as weight loss, with the humane euthanasia endpoints being >20% weight loss, observation of labored breathing, and severe lethargy (no response to manual stimulation). At 14 days post-infection, all remaining mice were euthanized via avertin overdose followed by cervical dislocation. As mice are less susceptible to infection with human H3N2 influenza viruses, we leveraged a mouse-adapted A/Switzerland/9715293/2013 virus, which has previously shown to productively infect mice and cause mortality ranging from 40 to 100% [32,33]. Using this virus, we did not observe sufficient mortality at doses of 10^4^, 10^5^, or 10^6^ FFU; therefore, we focused our efforts on assessing viral lung titers following challenge. For each H3N2 influenza challenge, five-to-six week old female DBA/2J mice (Jackson Laboratory, Bar Harbor, MA, USA) were used and administered a dose of 10^4^ FFU of the mouse-adapted A/Switzerland/9715293/2013 virus through an intranasal route (40 µL total, 20 µL each nostril) and mice were euthanized 3 days post-infection. After euthanasia, lungs from the mice were extracted, washed in PBS, and immediately homogenized with the gentleMACs dissociator machine (Miltenyi Biotech, San Jose, CA, USA) using the preset m_lung_02 setting, as previously described [34]. The homogenized lung tissue was spun down at 4000× *g* for 10 min, and the lung supernatant was collected and stored in the −80 °C until further use.

### 2.6. Mouse Experiments

Each immunization experiment was separated into 3 groups: HA + CpG, HA + VacSIM + CpG, and VacSIM + CpG. The delivery platform, VacSIM, was obtained from 3-D Matrix Medical Technology (Reference Number 621-014, Tokyo, Japan). The original VacSIM stock (2.5% RADA4 W/V) was diluted to 1.0% W/V in ddH_2_O. Before immunization, both HA antigen and CpG (vac-1826-1, InvivoGen, San Diego, CA, USA) were mixed with a 1:1 dilution of 1% VacSIM and 1X PBS (with the final concentration of VacSIM being 0.5% W/V) and transferred to a syringe for same-day immunization. For each immunization experiment, five-to-six-week-old female DBA/2J mice (*n* = 12 mice per group) (Jackson Laboratory) were immunized intramuscularly (both homologous 100 µL immunizations in the left leg muscle 4 weeks apart) with 3 µg of Y2 COBRA or NG2 COBRA protein. These immunizations were adjuvanted with 3 µg of CpG with or without the addition of VacSIM in a total of 100 µL (HA:CpG:VacSIM volume ratio of 1:1:2; 25 µL of 0.1 mg/mL HA, 25 µL of 0.1 mg/mL CpG, and 50 µL of 1.0% W/V VacSIM). As a negative control group, a subset of mice was immunized with just CpG and VacSIM. Mice were bled 1 day before immunization for baseline serum titers (Day −1 serum). Mice were bled again at 27 days post-prime for Day 27 serum, then boosted at day 28 post-prime with a homologous immunization. At 55 days post-prime, animals were bled for Day 55 serum and challenged with a respective H1N1 or H3N2 virus at 56 days post-prime. After blood collection, the serum was stored in the −80 °C freezer until further use. For the cocktail immunization experiment, five-to-six week old female DBA/2J mice (*n* = 12 mice per group) were immunized intramuscularly (both 100 µL immunizations in the left leg muscle 4 weeks apart) with 3 µg of Y2 and 3 µg of NG2. These immunizations were adjuvanted with 3 µg of CpG with or without the addition of VacSIM, to a total of 100 µL. As a negative control group, a subset of mice was immunized with just CpG and VacSIM. Serology readouts were the same as described above. For the single immunization experiment, five-to-six-week-old female DBA/2J (*n* = 12 mice per group) (Jackson Laboratory) were immunized intramuscularly (in the left leg muscle) with 10 µg of Y2 COBRA. These immunizations were adjuvanted with 10 µg of CpG with or without the addition of VacSIM. As a negative control group, a subset of mice was immunized with just CpG and VacSIM. Mice were bled 1 day before immunization for baseline serum titers (Day −1 serum) and again 27 days post-prime for Day 27 serum. These mice were subsequently challenged with a respective H1N1 virus 28 days post-prime.

### 2.7. Analysis of Lung Viral Titers

For determination of H3N2 lung viral titers, MDCK SIAT-1 cells were plated at a concentration of 4.8 × 10^6^ cells/mL (6 × 10^5^ cells per well) in 96-well plates and grown at 37 °C with 5% CO_2_ for 24 h, until confluent. The cells were washed twice with PBS. Following the wash, 50 µL of virus growth media (VGM: DMEM with L-glutamine (10-013-CM, Corning), 7.5% Bovine Serum Albumin (BSA) (BP9700100, Thermofisher), 1 µg /mL TPCK-Trypsin (20233, Thermofisher)) was added to each well, and the plate was incubated at 37 °C until ready for use. The collected lung supernatant from each mouse was diluted 10-fold in VGM across a 96-well plate. 50 µL of each dilution was added to the MDCK SIAT-1 cells and incubated at 37 °C for 2 h. After 2 h, an overlay was added to the cells (containing DMEM (MT90013PB, Corning), 0.1% BSA (BP9700100, Thermofisher), 1.2% Avicel ((11-101-5796, Thermofisher), 1 µg/mL TPCK-Trypsin (20233, Thermofisher), 2.4% cellulose (AAA1773036, Thermofisher)) and placed back in the 37 °C incubator overnight. The following day, the cells were observed for the presence of CPE and washed twice with PBS. The cells were then fixed for 30 min with 20% acetone/80% methanol at 4 °C, then subsequently washed. Following the wash, the plates were incubated with permeabilization buffer (glycine in 1X PBS (G46-500, Thermofisher), 5% Triton X-100 (AAA16046AE, Thermofisher) for 30 min at room temperature and washed 2× with 0.05% PBS-Tween-20. A mouse polyclonal antibody (FR-1217, IRR) targeting the nucleoprotein of the influenza virus was diluted 1:5000 in FRA ELISA buffer (10% heat-inactivated goat serum (16-210-072, Thermofisher, 0.01M PBS, 0.1% Tween-20 (AAJ20605AP, Thermofisher) for 1 h at room temperature and then washed 2× with 0.05% PBS-Tween-20. Goat anti-mouse IgG Fc-HRP secondary antibody (1030-05, Southern Biotech) diluted 1:4000 in FRA ELISA buffer, was added and incubated for 1 h at room temperature. The plates were washed 3× with 0.05% PBS-Tween-20. Afterwards, TrueBlue peroxidase (SeraCare^TM^, 5510-0030) was added for 15 min in the dark at room temperature. The plates were washed with ddH_2_O, allowed to dry, and read on a CTL ImmunoSpot machine. As a negative control, each plate contained a cell only control to ensure the cells were viable throughout the experiment.

### 2.8. Endpoint Titer Experiments

To determine the endpoint titers of the Day 0, 27, and 55 serums, enzyme-linked immunosorbent assays (ELISAs) were performed. 384-well plates were coated with an antigen (Y2 or NG2) diluted to 2 µg/mL in PBS at 4 °C overnight. The plates were washed 1× with ddH_2_O and blocked with 2% blocking buffer (2% nonfat dry milk powder (50-488-785, Thermofisher) dissolved in 0.05% PBS-Tween-20 with 2% goat serum (16-210-072, Thermofisher) added) for 1 h at 37 °C. The plates were washed 3× with ddH_2_O and diluted mouse serum was added and incubated for 1 h at 37 °C. The mouse serum was diluted three-fold from an initial 1:50 dilution in 1% blocking buffer (2% blocking buffer diluted 1:1 in PBS). The plates were washed 3× with ddH_2_O, and a goat anti-mouse IgG Fc-AP secondary antibody (1033-04, Southern Biotech), diluted 1:4000 in 1% blocking buffer, was added and incubated for 1 h at 37 °C. The plates were washed 5× with 0.05% PBS-Tween-20. P-Nitrophenyl phosphate (PNPP) substrate (34045, Thermofisher) was added to substrate buffer (1.0 M Tris base (BP152-500, Thermofisher), 0.5 mM MgCl_2_ BP214-500, Thermofisher)), pH 9.8) to a concentration of 1 mg/mL and added to the plates. The substrate was incubated for 30 min in the dark at room temperature, and then subsequently read at 405 nm on a BioTek plate reader (BioTek, Winooski, VT, USA).

### 2.9. Hemagglutination Inhibition Assays (HAIs)

Immunized mouse serum was treated with receptor-destroying enzyme II (RDE II, Senka Seiken, Toyko, Japan) to destroy nonspecific hemagglutination inhibitors within the serum. One volume of serum was diluted in three volumes of RDE II in PBS and incubated at 37 °C overnight. The following morning, the treated serum was heat-inactivated at 56 °C for 45 min, and then six volumes of PBS were added. Influenza viruses were titered to a 1:8 dilution, using the HA titer dilution determined prior. 50 µL of RDE-treated serum was diluted two-fold in PBS across a 96-well V-bottom plates for a total of 11 dilutions. 50 µL of titered virus (1:8 hemagglutination units per mL) was added to each serum dilution, and the wells were mixed and incubated at room temperature for 30 min. Following this, 50 µL of 1.0% turkey whole blood [16] (H1N1 viruses) or 1.0% guinea pig whole blood [35] (H3N2 viruses) was added to each dilution well, and the plates were incubated for 30 min. After 30 min, plates were read by the eye, where the well with the highest serum dilution that red blood cells were not agglutinated was measured as the HAI titer. We used a 1:40 titer as the definition of seroprotection as defined by the World Health Organization and the European Committee for Medicinal Products [36].

### 2.10. Quantification and Statistical Analysis

Two-way ANOVA was used to compare all endpoint serum titers between all vaccination groups using GraphPad Prism software version 10.3.1 (GraphPad, San Diego, CA, USA). One-way ANOVA was used for comparison of HAI titers between vaccination groups and for lung viral titers in the challenged NG2-immunized groups. Log-Rank (Mantel–Cox) was used to compare survival between all vaccination groups. Statistical significance was defined as follows: *, *p* ≤ 0.05, ** *p* ≤ 0.01; *** *p* ≤ 0.001; **** *p* ≤ 0.0001. Level of detection (LOD) for the reciprocal endpoint titers was identified as the lowest detectable dilution included on the plate (1:50). LOD for lung viral titers was identified as the lowest detectable number of FFU in the lowest dilution, which was 400 FFU. LOD for HAIs was identified as the lowest detectable dilution on the plate (1:5). Positive threshold cutoffs for reciprocal endpoint titers were determined as previously described by taking the OD405 of the negative control sample wells and adding 3× the standard deviation (OD405 + 3×STDEV), which gave a value of 0.25 as the cutoff.

## 3. Results

### 3.1. Vaccination with VacSIM and HA Increases Antigen-Specific IgG Antibody Titers

**Figure 1:**
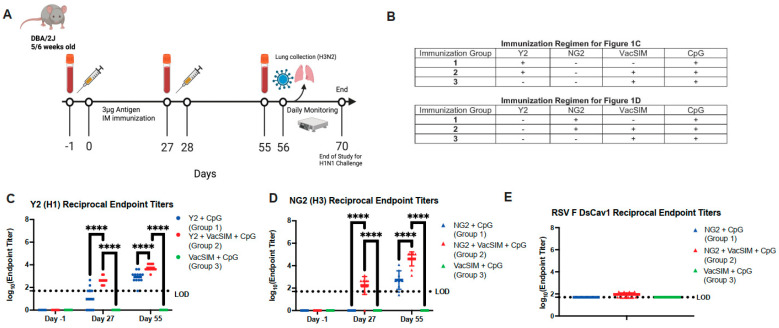
Antigen-specific serum antibody titers of immunized mice at Days −1, 27, and 55. (**A**) Mice were either immunized with the Y2 (H1) or NG2 (H3) COBRA HA proteins using a prime-boost regimen with 4 weeks between prime and boost. (**B**) Outline of the vaccination groups. Serum was collected from mice at three time points, Day −1 pre-prime, 27 days post-prime, and 55 days post-prime. This serum was analyzed by ELISA screening for (**C**) Y2 (H1) HA-immunized mice and (**D**) NG2 (H3) HA-immunized mice (*n* = 12 mice per group). (**E**) D55 NG2 serum was tested by ELISA against RSV F DsCav1 protein to determine serum titers against the foldon trimerization domain. A minimum baseline absorbance at 405 nm of 0.25 was considered a positive binding signal. Limit of detection for reciprocal endpoint titers was a 1:50 serum dilution with samples below this line showing no detectable serum titers. Circles represent Y2 immunized mice, and triangles represent NG2 immunized mice. ****, *p* ≤ 0.0001, statistics obtained by two-way ANOVA test.

To determine the effect of VacSIM on COBRA HA vaccination, DBA/2J mice were immunized intramuscularly (IM) in a prime-boost regimen with 3 µg of either Y2 (H1 HA from 2014 to 2016) or NG2 (H3 HA from 2016 to 2018) COBRA proteins, with or without VacSIM, in the presence of the TLR9 agonist adjuvant, CpG ODN 1826 (Figure 1A,B). Mice were bled at day −1 pre-prime, 27 days post-prime, and 55 days post-prime to obtain serum for serology studies. Serum was then evaluated to determine antigen-specific IgG antibody titers by enzyme-linked immunosorbent assay (ELISA) against the homologous immunizing antigen, either Y2 or NG2 (Figure 1C,D). At 27 days post prime, mice immunized with HA + VacSIM + CpG had significantly increased antibody titers compared to mice immunized with HA + CpG, independent of the HA antigen (Figure 1C,D). This trend was consistent at 55 days post-prime where vaccination with HA + VacSIM + CpG led to a ~1 to 2 log increase in antigen-specific antibody titers for Y2 and NG2, respectively, in comparison to vaccination with HA + CpG (Figure 1C,D). To understand if vaccine-elicited antibodies were targeting the foldon trimerization domain in the HA antigen, Day 55 serum from NG2-immunized mice was tested against an irrelevant RSV F protein (DS-Cav1) [37,38] containing a foldon trimerization domain, and minimal serum cross-reactivity was observed, suggesting the majority of the response was HA-specific (Figure 1E).

### 3.2. Vaccination with VacSIM and HA Increases HAI Titers Across Seasonal Influenza Viruses

**Figure 2:**
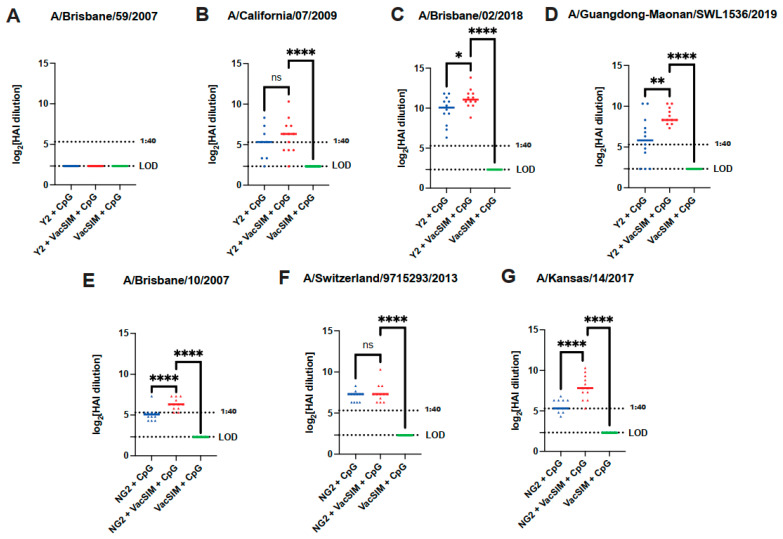
HAI titers of Day 55 serum from Y2- and NG2-immunized mice against a panel of influenza viruses. (**A**–**D**) Y2 immunized mouse serum was incubated with turkey RBCs and HAI activity was measured against a panel of H1N1 viruses (*n* = 12 mice per group). (**E**–**G**) NG2-immunized mouse serum was incubated with guinea pig RBCs and HAI activity was measured against a panel of H3N2 viruses (*n* = 12 mice per group). Titers are represented as log2-transformed reciprocal dilutions. The upper dotted line represents the 1:40 titer, typically defined as the threshold for seroprotection. Limit of detection for HAI titers was a 1:5 dilution with samples below the line showing no detectable HAI titers. Circles represent Y2 immunized mice, and triangles represent NG2 immunized mice. ns, nonsignificant; *, *p* ≤ 0.05, ** *p* ≤ 0.01; **** *p* ≤ 0.0001, statistics obtained by ordinary one-way ANOVA test.

Hemagglutination inhibition assays (HAIs) are an important benchmark in influenza vaccination studies to determine whether vaccine-elicited antibodies can block the binding of the influenza virus to the sialic acid receptors on red blood cells. The presence of receptor-binding domain antibodies bound to the virus will stop binding of the virus to red blood cells, decreasing agglutination [39]. To determine whether the vaccine-elicited serum antibodies could block influenza virus receptor binding, HAIs were performed against panels of H1N1 or H3N2 influenza viruses. Serum from mice immunized with the Y2 (H1 HA) COBRA HA protein was tested against a panel of four H1N1 influenza viruses, one pre-2009 pandemic strain, A/Brisbane/59/2007, and three recent 2009 pandemic-like H1N1 viruses, A/California/07/2009, A/Brisbane/02/2018 and A/Guangdong-Maonan/SWL1536/2019 (Figure 2A–D). Vaccination with Y2 + VacSIM + CpG led to an increase in HAI activity for A/Brisbane/02/2018 and A/Guangdong-Maonan/SWL1536/2019, but not for A/California/07/2009, in comparison to Y2 + CpG (Figure 2B–D). Furthermore, no HAI activity was observed against the pre-2009 pandemic H1N1 virus A/Brisbane/59/2007 (Figure 2A). Similarly, serum from mice immunized with the NG2 (H3 HA) COBRA HA protein was tested against a panel of three H3N2 influenza viruses, A/Brisbane/10/2007, A/Switzerland/9715293/2013, and A/Kansas/14/2017 (Figure 2E–G). A significant increase in HAI activity against the H3N2 viruses A/Brisbane/10/2007 and A/Kansas/14/2017 was observed for mice immunized with NG2 + VacSIM + CpG in comparison to NG2 + CpG-vaccinated mice; however, there was no significant difference observed for A/Switzerland/9715293/2013. Overall, immunization with VacSIM increased vaccine-elicited antibody titers and serum HAI activity across multiple influenza viruses.

### 3.3. Vaccination with VacSIM and HA Improves Protection Against Influenza Infection

**Figure 3:**
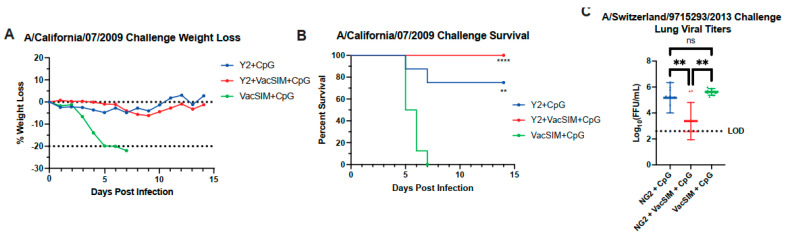
Protective efficacy of Y2 or NG2 immunization after viral challenge. Y2-immunized mice were challenged with 10^3^ FFU of A/California/07/2009 56 days post-prime. (**A**) These mice were monitored for survival over the course of 14 days, monitoring weight loss, with 20% weight loss being the humane euthanasia endpoint. (**B**) Percent survival was calculated for each challenge group (*n* = 8 mice per group). **, *p* ≤ 0.01; ****, *p* ≤ 0.0001 (compared to the VacSIM + CpG immunization group), statistics obtained by Log-Rank (Mantel–Cox) test. (**C**) NG2-immunized mice were challenged with a sublethal dose of A/Switzerland/9715293/2013 56 days post-prime. Mice were euthanized 3 days post-infection, and lungs were collected for analysis of lung viral titers (*n* = 8 mice per group). Limit of detection for lung viral titers was 400 FFU with samples below this line showing no detectable lung viral titers. Circles represent Y2 immunized mice, and triangles represent NG2 immunized mice. ns, nonsignificant; **, *p* ≤ 0.01, statistics obtained by ordinary one-way ANOVA test.

To determine whether the addition of VacSIM could increase protection observed after influenza challenge, immunized mice were challenged 56 days post-prime with a respective influenza virus. The Y2 (H1 HA)-immunized mice were challenged with 10^3^ FFU/mouse of A/California/07/2009 and were monitored over the course of 14 days for weight loss and the presence of labored breathing and lethargy. All mice immunized with Y2 HA protein had minimal weight loss, demonstrating that vaccination with this antigen protected the mice from challenge as previously observed (Figure 3A) [17]. Mice immunized with Y2 + VacSIM + CpG had 100% survival in comparison to 75% survival within the Y2 + CpG group (Figure 3B). NG2 (H3 HA)-immunized mice were challenged with a mouse-adapted A/Switzerland/9715293/2013 virus. Mice were euthanized 3 days post infection for analysis of lung viral titers. Mice vaccinated with NG2 + VacSIM + CpG had a significant decrease in lung viral titers in comparison to the NG2 + CpG and VacSIM + CpG group, with 6/8 mice showing no virus within the lungs at the limit of detection (LOD) (Figure 3C). Surprisingly, mice immunized with NG2 + CpG had no significant difference in lung viral titers in comparison to those mice immunized with VacSIM + CpG, demonstrating that immunization with NG2 + CpG could not prevent replication of virus within the lungs. Through both the H1N1 and H3N2 challenge studies, it was observed that vaccines including Y2 and VacSIM increased protection following influenza challenge, and vaccines including NG2 and VacSIM decreased lung viral titers following challenge. 

### 3.4. Vaccination with VacSIM and an HA Cocktail Increases Antigen-Specific IgG Antibody Titers Against Both Antigens

**Figure 4:**
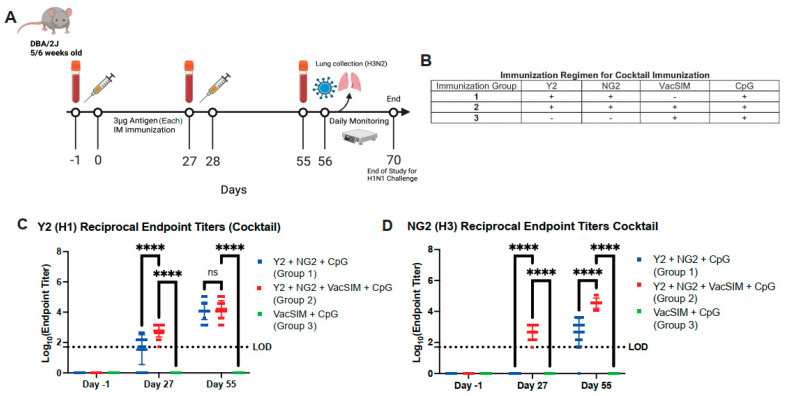
Antigen-specific antibody titers of cocktail immunized mouse serum (H1 and H3) from Days −1, 27, and 55. (**A**) Mice were immunized following a prime-boost regimen containing both the Y2 (H1) HA and NG2 (H3) HA. (**B**) Outline of cocktail vaccination groups. Serum was collected from these mice at three time points: Day −1 pre-prime, 27 days post-prime, and 55 days post-prime. This serum was analyzed through ELISA screening against both antigens, (**C**) Y2 HA and (**D**) NG2 HA (*n* = 12 mice per group). A minimum baseline absorbance at 405 nm of 0.25 was considered a positive binding signal. Limit of detection for reciprocal endpoint titers was a 1:50 serum dilution with samples below this line showing no detectable serum titers. Squares represent Y2 and NG2 immunized mice. ns, nonsignificant; ****, *p* ≤ 0.0001, statistics obtained by two-way ANOVA test.

We next determined if immunization with a cocktail of both Y2 (H1 HA) and NG2 (H3 HA) could demonstrate similar protection against influenza virus challenge. Mice were immunized intramuscularly with 3 µg of both Y2 and NG2, following the regimen outlined in Figure 4A,B. Serum was collected at −1 day pre-prime, 27 days post-prime, and 55 days post-prime and was analyzed through ELISA to determine the antigen-specific antibody titers against both Y2 and NG2 antigens. Antigen-specific antibody titers were significantly increased for Y2 at day 27 post-prime and NG2 at days 27 and 55 post-prime for the Y2 + NG2 + VacSIM + CpG-immunized groups in comparison to Y2 + NG2 + CpG-immunized groups (Figure 4C,D). This shows that an immunization regimen containing VacSIM and multiple antigens can lead to a significant increase in antigen-specific antibody titers against all antigens. 

### 3.5. Vaccination with VacSIM and HA Cocktail Improves Protection Against Both H1N1 and H3N2 Influenza Challenge

**Figure 5:**
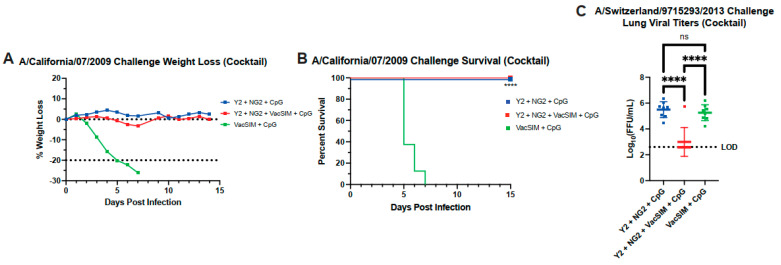
Protective efficacy of cocktail immunization (Y2 and NG2) against H1N1 and H3N2 influenza challenge. A subset of these cocktail immunized mice was challenged with 10^3^ FFU of A/California/07/2009. (**A**) These mice were monitored for 14 days, monitoring weight loss, with 20% weight loss being the humane euthanasia cutoff. (**B**) Percent survival was calculated for each challenge group (*n* = 8 mice per group) ****, *p* ≤ 0.0001 (compared to the VacSIM + CpG immunization group), statistics obtained by Log-Rank (Mantel–Cox) test. (**C**) A second subset of these cocktail immunized mice was challenged with a sublethal dose of A/Switzerland/9715293/2013 56 days post-prime. These mice were euthanized 3 days post infection, and lungs were collected for lung viral titers (*n* = 8 mice per group). Limit of detection for lung viral titers was 400 FFU with samples below this line showing no detectable lung viral titers. Squares represent Y2 and NG2 immunized mice. ns, nonsignificant; ****, *p* ≤ 0.0001, statistics obtained by ordinary one-way ANOVA test.

To determine if the cocktail immunization with VacSIM containing Y2 and NG2 could protect against both an H1N1 and H3N2 influenza challenge, immunized mice were challenged 56 days post-prime with either an H1N1 virus or H3N2 virus as in Figure 4A. A subset of mice was challenged with a lethal dose of A/California/07/2009 (H1N1) and were monitored for weight loss, labored breathing, and lethargy over the course of 14 days. Mice in both the Y2 + NG2 + VacSIM + CpG group and Y2 + NG2 + CpG group had minimal weight loss over the course of 14 days (Figure 5A). Similarly, both the Y2 + NG2 + VacSIM + CpG group and Y2 + NG2 + CpG group had 100% survival (Figure 5B). A second subset of mice was challenged with A/Switzerland/9715293/2013 and were euthanized 3 days post-infection for analysis of lung viral titers. Mice vaccinated with Y2 + NG2 + VacSIM + CpG had a significant decrease in lung viral titers in comparison to the Y2 + NG2 + CpG and VacSIM + CpG groups, with 7/8 mice having no detectable virus (Figure 5C). Immunization with Y2 + NG2 + CpG led to comparable levels of virus in the lungs compared to the VacSIM + CpG-immunized mice (Figure 5C). These data together demonstrate that addition of VacSIM within a cocktail immunization containing multiple antigens facilitates similar protection against lethal H1N1 challenge as that without VacSIM but decreases lung viral titers against H3N2 challenge.

### 3.6. Single Vaccination with VacSIM and Y2 Increases Antigen-Specific Antibody Titers and Protects Against Lethal H1N1 Infection

**Figure 6:**
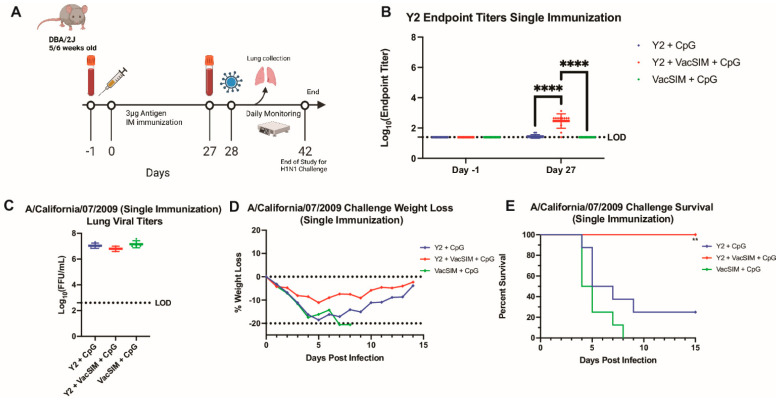
Protective efficacy of single Y2 immunization against lethal H1N1 influenza challenge. (**A**) Mice were immunized with 10 µg of the Y2 (H1) HA antigen using a single prime regimen. (**B**) Serum was collected from these mice at 2 time points: Day −1 pre-prime and 27 days post-prime. This serum was analyzed by ELISA to determine the antigen-specific antibody titers against the Y2 antigen. At Day 28, mice were challenged with 10^3^ FFU of A/California/07/2009. Limit of detection for reciprocal endpoint titers was a 1:50 serum dilution with samples below this line showing no detectable serum titers. ****, *p* ≤ 0.0001, statistics obtained by two-way ANOVA test. (**C**) A subset of these infected mice was taken down 3 days post infection, and lungs were collected to determine the lung viral titers (*n* = 4 mice per group). Limit of detection for lung viral titers was 400 FFU with samples below this line showing no detectable lung viral titers. (**D**) A second subset of mice was monitored for 14 days with 20% weight loss being the humane euthanasia cutoff. (**E**) Percent survival was calculated for each challenge group (*n* = 8 mice per group). Diamonds represent Y2 immunized mice after single immunization. **, *p* ≤ 0.01 (compared to the Y2 + CpG immunization group), statistics obtained by Log-Rank (Mantel–Cox) test.

As vaccination with VacSIM led to significantly increased antigen-specific antibody titers at day 27 post-prime for the Y2 COBRA HA protein, but all Y2-immunized groups had high survival with or without VacSIM, we next determined if one immunization with Y2 + VacSIM + CpG could improve protection against a lethal influenza challenge compared to Y2 + CpG-immunized mice. Mice were immunized once intramuscularly with 10 µg of Y2 (H1 HA) (Figure 6A). Consistent with what was observed previously, vaccination with Y2 + VacSIM + CpG led to a significant increase in antigen-specific antibody titers at day 27 after one immunization in comparison to mice vaccinated with Y2 + CpG (Figure 6B). These immunized mice were then challenged with a lethal dose of A/California/07/2009 (H1N1) and were either monitored for survival for 14 days or euthanized 3 days post infection to determine the lung viral titers. Lungs collected from each group had high levels of lung viral titers, with no difference observed in any of the groups (Figure 6C). Similarly, mice in all immunization groups had weight loss over the course of 14 days after challenge (Figure 6D), with the surviving mice recovering from disease and trending towards their original weight at the end of 14 days. However, when looking at overall survival, mice vaccinated with Y2 + VacSIM + CpG had 100% survival against H1N1 challenge, whereas mice vaccinated with Y2 + CpG had only 25% survival (Figure 6E). Overall, although mice immunized with Y2 + VacSIM + CpG presented with weight loss and had high lung viral titers, these mice had complete protection against mortality in comparison to the Y2 + CpG-immunized mice.

## 4. Discussion

Due to the low efficacy of seasonal influenza vaccines from year to year, there is a need to develop new strategies to circumvent this problem and induce a broadened and more robust immune response, capable of providing longer-lasting protection. Platforms facilitating extended antigen release may improve the longevity of antigen within the host leading to improved immune responses [19,40]. In this study, we aimed to determine the effect of an extended delivery platform, VacSIM, on the vaccine-elicited serum antibody response and functionality against two influenza COBRA antigens, Y2 and NG2. Previous studies have shown that vaccination with Y2 (H1 HA) and NG2 (H3 HA) lead to a broader humoral response that protects against subsequent challenge [16]. To test whether addition of VacSIM could improve the humoral response to COBRA HA vaccination, we assessed antibody titers to the immunizing antigens. Immunization with HA + VacSIM + CpG increased serum antibody titers in comparison to immunization with HA + CpG at both 27 days post-prime and 55 days post-prime. This trend was consistent between vaccination with both Y2 and NG2, as well as with previous work demonstrating the use of different slow delivery platforms, such as use of osmotic pumps or escalating dose over a period of time [23,41].

Knowing that addition of VacSIM increased the quantity of vaccine-elicited antibodies, we determined if VacSIM could increase the breadth and functionality of these antibodies. Previous work has shown that vaccination with Y2 and NG2 alone led to an increase in HAI activity across a panel of influenza viruses [16,17]. Here, we show that vaccination with HA + VacSIM + CpG led to an increase in HAI titers against a subset of H1N1 and H3N2 influenza viruses in comparison to mice vaccinated with HA + CpG. Following Y2 immunization, we observed no HAI activity against the pre-2009 pandemic strain, A/Brisbane/07/2007. One possible explanation for this is that Y2 was designed using influenza virus sequences from the years 2014–2016 and is thus unable to effectively elicit antibodies to pre-2009 pandemic-like H1N1 influenza viruses as pre-pandemic HA proteins contain mutations that make it unrecognizable by an immune response towards a post-pandemic HA vaccine [42]. These results are consistent with previous work with serum collected from mice immunized daily with an H1N1/2009-derived whole inactivated virus [41]. Similarly to this study, the authors observed an increase in HAI activity against post-pandemic H1N1 viruses, but no activity against pre-pandemic H1N1 viruses [41].

For H3N2 vaccination, there was no significant difference in HAI titers between NG2 + VacSIM + CpG and NG2 + CpG-vaccinated groups against A/Switzerland/9715293/2013. These results are surprising, as we observed mice vaccinated with NG2 + VacSIM + CpG had a significant reduction in lung viral titers 3 days post challenge with A/Switzerland/9715293/2013 in comparison to mice immunized with NG2 + CpG. However, HAIs only address vaccine-elicited antibodies targeting the receptor binding domain. While the receptor binding domain antibodies may have comparable titers, there are possibly more antibodies that function independently of receptor binding. To address these questions, further research should assess the functionality of these vaccine-elicited antibodies through neutralization assays and by studying Fc-effector functions, which consider multiple alternative ways to target the virus.

With the increased quantity and functionality of these vaccine-elicited antibodies, we determined if the addition of VacSIM could increase the quality of these vaccine-elicited antibodies in a protection study. Previous work has shown that vaccination with Y2 and NG2 with adjuvant protected against subsequent influenza infection [16,43]. For the Y2 immunized mice, there was complete protection against a lethal H1N1 infection for the Y2 + VacSIM + CpG group. Similarly, for the NG2 study, mice immunized with NG2 + VacSIM + CpG group had significantly less lung viral titers compared to NG2 + CpG vaccinated mice, demonstrating that immunization with just antigen does not fully protect against viral replication.

We next tested if the same results would be observed with an immunization regimen containing both Y2 and NG2 COBRA HA proteins. Here, similar trends were seen after vaccination with Y2 + NG2 + VacSIM + CpG for total antibody titers, protection against H1N1 influenza challenge, and for reduced H3N2 lung viral titers in comparison to groups immunized with Y2 + NG2 + CpG. These data are important because it shows that inclusion of two different antigens within one vaccination led to an equal distribution of the humoral immune response to both antigens, which was comparable to immunization with single antigens.

Since mice vaccinated in a prime-boost regiment with Y2 + CpG had high survival following H1N1 influenza challenge, and no difference was observed compared to mice vaccinated with Y2 + VacSIM + CpG, we aimed to determine if a single immunization of Y2 + VacSIM + CpG could increase protection after a subsequent H1N1 influenza infection. A single immunization with Y2 + VacSIM + CpG led to a significant increase in survival against lethal challenge compared to mice vaccinated with Y2 + CpG but did not reduce lung viral titers. This particular study is important because one of the greatest burdens of influenza vaccination is the need for an annual vaccine with improved protection, which could potentially be induced by platforms facilitating a more robust response.

Future work in this project aims to elucidate the mechanism behind the VacSIM platform that facilitates the increase in vaccine-elicited antibodies. Extended delivery of antigen could potentially mimic host infection [40,41]. During primary infection, the host tissues are exposed to antigens for sustained periods, up to weeks or months, whereas immunization intramuscularly with a bolus of protein antigen is typically cleared by the immune system within days [19,23]. Previous work using different slow delivery techniques have shown that extended availability to the antigen leads to increased antibody titers, increased germinal center reactions, and a more diverse array of antibodies, potentially through an increase in somatic hypermutation [23,41]. While we do not have direct data on the mechanism of VacSIM and how it allows for this increased humoral immune response, we are actively exploring this mechanism of action. Alternatively, as the goal of this paper was to determine if the breadth of antibody response could be increased using VacSIM, future work should focus deeper on the cellular immune response to see if VacSIM also affects cellular immunity. While this study focused on one adjuvant, CpG, due to its small size and previous use within the VacSIM platform, future work should also test different adjuvants within the VacSIM platform to examine if the humoral and/or cellular immune response is enhanced. Lastly, while mouse models are commonly used in influenza vaccination studies because they are able to support both H1N1 and H3N2 influenza virus replication, a ferret model would be a better model to study virus transmission. Future work should include this ferret model to show the translational relevance of the findings to humans.

## Data Availability

The data presented in this study will be available in the Statistical, Data Management, and Coordination Center through CIVICs (https://www.niaidcivics.org/statistical-data-management-and-coordination-center-sdmcc/, accessed on 26 July 25). This data will further be accessible on NIH IMMPORT.

## Abbreviations

HA	hemagglutinin
NA	neuraminidase
VacSIM	Vaccine Self-Assembling Immune Matrix
COBRA	computationally optimized broadly reactive antigen
HAI	hemagglutination inhibition assay
